# One-pot hydrothermal synthesis of FeNbO_4_ microspheres for effective sonocatalysis

**DOI:** 10.1039/d3nj05239g

**Published:** 2024-03-11

**Authors:** Min He, Defa Li, Yu Liu, Taohai Li, Feng Li, Javier Fernández-Catalá, Wei Cao

**Affiliations:** a College of Chemistry, Key Lab of Environment Friendly Chemistry and Application in Ministry of Education, Xiangtan University China fengli_xtu@hotmail.com; b Nano and Molecular Systems Research Unit, University of Oulu P.O. Box 3000 FIN-90014 Finland wei.cao@oulu.fi; c Inorganic Chemistry Department, Materials Science Institute, University of Alicante Ap. 99 Alicante 03080 Spain

## Abstract

FeNbO_4_ sonocatalysts were successfully synthesized by a simple hydrothermal route at pH values of 3, 5, 7, 9 and 11. The catalysts were characterized by XRD, XPS, TEM, SEM, N_2_ adsorption and DRS to analyse the effect of pH parameters on the physicochemical properties of the materials during hydrothermal synthesis. The sonocatalytic activity of FeNbO_4_ microspheres was evaluated by using acid orange 7 (AO7) as the simulated contaminant. The experimental results showed that the best sonocatalytic degradation ratio (97.45%) of organic dyes could be obtained under the conditions of an initial AO7 concentration of 10 mg L^−1^, an ultrasonic power of 200 W, a catalyst dosage of 1.0 g L^−1^, and a pH of 3. Moreover, the sonocatalysts demonstrated consistent durability and stability across multiple test cycles. After active species capture experiments and calculation of the energy band, a possible mechanism was proposed based on the special Fenton-like mechanism and the dissociation of H_2_O_2_. This research shows that FeNbO_4_ microspheres can be used as sonocatalysts for the purification of organic wastewater, which has a promising application prospect.

## Abstract 2

FeNbO_4_ can effectively sonocatalytically remove AO7.

## Graphical abstract

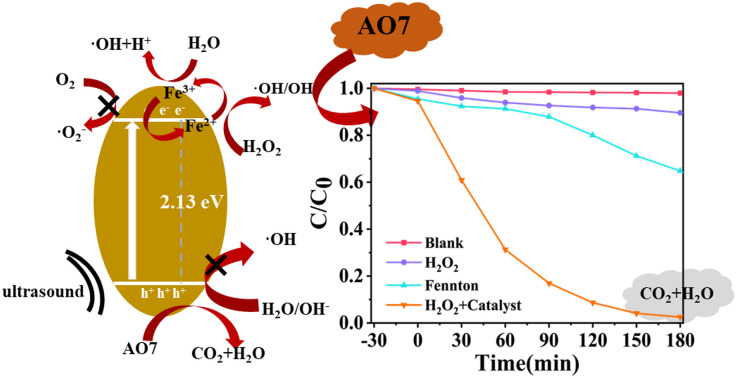

## 1. Introduction

In recent years, industrialization has brought unprecedented improvements to human life, but also caused serious harm to the environment. Dye wastewater is one of the main pollutants discharged in industrial production.^1^ Currently, the annual production of dyes in the world exceeds 1.3 million tons. About 100 000 of these dyes are used in different industrial products, resulting in the discharge of large quantities of dye pollutants into the production wastewater of industries.^2–4^ The discharge of these organic dyes into the aquatic environment may be mutagenic, carcinogenic and teratogenic, posing a serious threat to waterbodies and human health.^5,6^ Azo dyes are an important component in dye wastewater.^7^ Among them, acid orange 7 (AO7) is a widely used anionic azo dye, which is not only highly aromatic and toxic to microorganisms, but also has azo groups that effectively resist biodegradation.^8,9^ Since AO7 is produced in large quantities, widely used, and difficult to degrade, it is imperative to study how to completely degrade it into harmless substances.

Advanced oxidation processes (AOPs) have been proved to be effective in degrading various pollutants.^10–12^ Sonocatalytic degradation technology is a type of AOP that relies on the simultaneous presence of an appropriate semiconductor catalyst and ultrasonic radiation.^13^ The hydroxyl radicals (˙OH) generated during the sonocatalytic process can more effectively convert organic pollutants in water into carbon dioxide, water, and inorganic ions, or less toxic and more easily degradable substances.^14–17^ However, the degradation of organic wastewater by single ultrasonic irradiation is not satisfactory. Studies have shown that the combination of two or more treatment techniques is more economical and effective than the single ultrasonic degradation technology.^18,19^ The combination of heterogeneous Fenton-like catalysis and sonocatalysis is currently being investigated and is considered another possible way to increase free radical production in the system, with a great enhancement in terms of improving the degradation.^17,18^

So far, a series of metal complex oxide niobates (EuNbO_4_, BiNbO_4_, AgNbO_3_, and FeNb_2_O_6_) have been discovered and applied in the fields of photocatalysis, electrocatalysis, and electrolysis.^20–22^ However, there are few reports on the sonocatalytic activity of niobates.^23–25^ FeNbO_4_ is a metal oxide type of ABO_4_ with good magnetic and electrical properties, unique narrow band gaps, and excellent photocatalytic properties.^26,27^ It has recently attracted attention for its potential applications in photocatalysis, photodetectors, and gas sensors.^28–30^ However, the conditions for the synthesis of niobates are relatively harsh, and the traditional high-temperature solid-phase method for the synthesis of FeNbO_4_ requires temperatures of more than 1000 °C.^31^ The samples obtained by this method are characterized by large particle size, low crystallinity, and phase mixing, which can lead to the low catalytic activity of niobates, and greatly limit their application in catalytic water treatment.^30,32^ Therefore, the scientific community has been developing new synthesis strategies in recent years. Up to now, several attempts to reduce the synthesis temperature of FeNbO_4_ have been reported. Recently, Devesa *et al.*^33^ reported the synthesis of FeNbO_4_ with monoclinic and orthorhombic structures by the sol–gel method. The nanomaterials prepared by this method can be mixed up to the molecular level, but the secondary phase of Fe_2_O_3_ has been present with the increase of the heat treatment temperature from 400 °C to 1200 °C. Wang *et al.*^34^ prepared FeNbO_4_ nanorods with a diameter of 100 nm and a length of 1–3 μm by electrostatic spinning. The advantage of this method is that adjustable fibers can be prepared, but the yield is low. Shim *et al.*^35^ produced FeNbO_4_ nanopowder with a smaller particle size and a larger specific surface area under hydrothermal reaction conditions at 250 °C. This method avoids the problems of oversized grains, crystal defects, and the introduction of heteroatoms caused by the calcination method at high temperatures. However, a high hydrothermal temperature places stringent requirements for the preparation equipment. Ahmed *et al.*^36^ obtained the finer and uniformly distributed FeNbO_4_ by co-precipitation of reaction regents of ferric nitrate and ammonium niobate oxalate hydrate. The process was followed by annealing at 1100 °C for 6 h. Besides the niobate, the accompanied NbO_2_ phase was also detected. In addition, almost all of the above syntheses of FeNbO_4_ are based on the more expensive niobium pentachloride and requirements of heat treatments above 900 °C. Therefore, the study of new methods to achieve simple, economical and controllable synthesis reactions at low temperatures or the improvement of existing methods is an urgent need for the application of this material. At present, there is no report on the sonocatalytic activity of FeNbO_4_. The FeNbO_4_ metal complex oxide semiconductor is expected to be applied to degrade pollutants in the field of acoustic catalysis.

Based on the above discussion, FeNbO_4_ microspheres were prepared in this study by a simple hydrothermal method (200 °C) under different pH conditions, using low temperatures and non-toxic reagents. The FeNbO_4_ microspheres were used as Fenton-type catalysts for the first time to degrade acid orange 7 (AO7) under ultrasonication (US) with H_2_O_2_ as the oxidant. In this work, the effects of catalyst, catalyst dosage, pollutant pH, and pollutant concentration on the performance of degradation of AO7 were investigated. The results show that FeNbO_4_ particles have an effective synergistic effect in the presence of ultrasonic radiation and H_2_O_2_. In addition, the possible sonocatalytic degradation mechanism of AO7 in the presence of FeNbO_4_ is preliminarily proposed.

## 2. Experimental

### 2.1 Materials

Niobium oxalate (C_10_Nb_2_O_20_) was sourced from Ningxia Dongfang Tantalum Industry Co., Ltd. Ferrous sulfate (FeSO_4_·7H_2_O), sodium hydroxide (NaOH) and isopropyl alcohol (IPA) were acquired from Shantou Xilong Co., Ltd. Potassium iodide (KI) was purchased from Tianjin Kemiou Chemical Reagent Co., Ltd. 1.4-Benzoquinone (BQ) was supplied by Shanghai Macklin Co., Ltd. Anhydrous ethanol (EtOH) and hydrogen peroxide (H_2_O_2_) were obtained from Hunan Huihong Reagent Co., Ltd. All reactants were used as received, without any further purification.

### 2.2 Synthesis of FeNbO_4_

FeNbO_4_ was synthesized by a simple hydrothermal method. Typically, 0.70 g of FeSO_4_·7H_2_O was dissolved in 5 mL of deionized water and named solution A. 2.69 g of C_10_Nb_2_O_20_ was mixed with 40 mL of deionized water and stirred for 30 min, and the solution was named solution B. Then, solution A was slowly added to solution B and stirred for an hour. Afterwards, the pH of the solutions was adjusted to several pH values (3, 5, 7, 9, 11) using 5 M NaOH solution; the solutions were transferred to a 50 mL Teflon reactor (autoclave) and heated at 200 °C for 24 h. After hydrothermal treatment, the autoclave was cooled to room temperature. The mixtures obtained were centrifuged (8000 rpm) to collect the solid materials and they were washed three times with DI water and three times more with anhydrous EtOH in order to clean the samples from some impurities obtained during the synthetic process. The solid obtained was dried using an oven at 70 °C for 24 h. The obtained samples were denoted as FN3, FN5, FN7, FN9, and FN11, respectively, following the pH values.

### 2.3 Characterization

The phase purity of sample powders was determined by X-ray powder diffraction (XRD, Bruker D8 ADVANCE, Germany) using the Cu Kα radiation (*λ* = 0.154 nm) at 40 mA and 40 kV and over the 2*θ* range from 5° to 80°. The morphology of the sonocatalysts was observed using a scanning electron microscope (SEM, JMS-6610LV, Japan) and a transmission electron microscope (TEM, JEM-2010, Japan). X-ray photoelectron spectroscopy (XPS, Escalab 250Xi, USA) was used to analyze the chemical valence of sample powders. The optical properties of the samples were analyzed using a UV-visible spectrophotometer (DRS, UV-2550, Japan) with BaSO_4_ powder as the reference calibration standard. Nitrogen adsorption–desorption isotherms were recorded using a nitrogen adsorption apparatus (TRISTAR II 3020, USA). Before the analysis, the samples were evacuated for 2 h at 150 °C. An electrochemical Workstation (Model CHI660E, China) was used to perform Mott–Schottky (MS) analysis. The absorbance of dyes was recorded using a UV-vis spectrophotometer (Carry 60, Agilent Company, USA).

### 2.4 Sonocatalytic test

The sonocatalytic activity of FeNbO_4_ was evaluated using acid orange 7 (AO7) as a pollutant. 50 mg of FeNbO_4_ and 1 mL of 30% H_2_O_2_ were added into 50 mL of AO7 solution with a concentration of 10 mg L^−1^ in a glass beaker. Before ultrasonic irradiation, the suspension was placed in the dark and magnetically stirred for 30 min to reach the adsorption–desorption equilibrium. Then, the degradation of the pollutant was performed in the 200 W ultrasonic bath for 180 min. The catalytic test was carried out in the dark environment to eliminate the effects of light irradiation. After the starting of sonication every 30 min, 3 mL of suspensions was sampled and centrifuged at 15 000 rpm for 5 min and separated the liquid from the solid. The liquid sample was measured using a UV-vis spectrophotometer (Carry 60, Agilent Company, USA) in the wavelength range of 200–800 nm to follow the pollutant degradation. The efficiency of degradation (*R*) was calculated by *R* = [(*C*_0_ − *C*_t_)/*C*_0_] × 100%, where *C*_0_ is the AO7 concentrations before irradiation (after adsorption equilibrium) and *C*_t_ (mg L^−1^) is the AO7 concentrations at certain time. Additionally, a blank test without sonication irradiation was performed under the same experimental conditions. To study the stability of the sample, after the reaction, the sample was recovered for performing cycling experiments. After each cycle, the catalyst was separated from the suspension by centrifugation (15 000 rpm). Then, the used catalyst was soaked in ethanol for at least 12 h to remove AO7 and others deposited on the surface of samples. Finally, the catalyst was collected by centrifugation (15000 rpm) and washed with deionized water and ethanol, dried in a vacuum at 70 °C overnight, and continued for a new cycle.

To explore the role of active species in the sonocatalysis process, the experiments of radical capture were carried out. The influence of active species to the photocatalytic degradation of tetracycline was investigated by adding 1 mM of isopropyl alcohol (IPA), potassium iodide (KI), and 1,4-benzoquinone (BQ) as scavengers to detect the hydroxyl radical (˙OH), hole (h^+^) and superoxide radical (˙O_2_^−^), respectively, before the photodegradation experiment.

## 3.Results and discussion

### 3.1 Characterization of FeNbO_4_

**Fig. 1:**
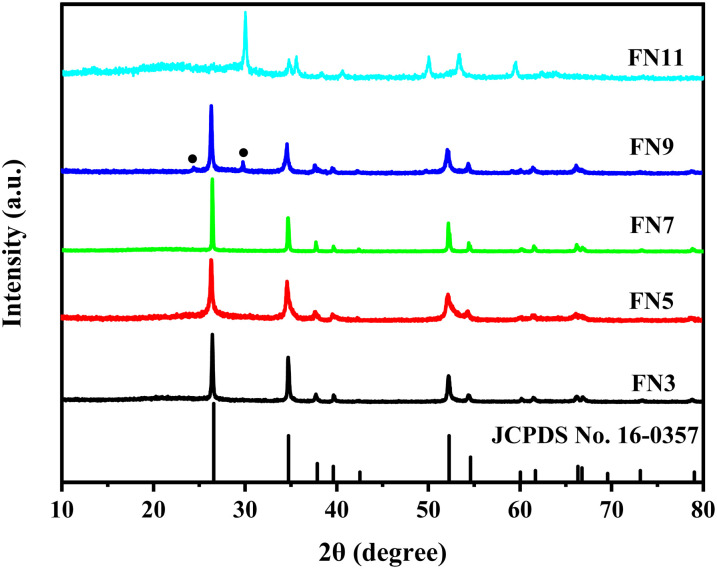
XRD patterns of FeNbO_4_ samples. • Indicates the (012) and (220) crystallographic plane positions of Fe_2_O_3_ (JCPDS No. 33–0664).

The crystal structures of FeNbO_4_ samples (FN3, FN5, FN7, FN9, and FN11) prepared by hydrothermal synthesis using different pH conditions were characterized by XRD analysis. Fig. 1 shows that all the samples prepared in this work have crystallinity. The samples of FN3, FN5, FN7, and FN9 showed diffraction peaks at 2*θ* degrees of 26.5°, 34.7°, 37.8°, 39.7°, and 52.3°, which is corresponding with the characteristic peaks of (110), (101), (200), (111), and (211) lattice planes of the tetragonal FeNbO_4_ (JCPDS No. 16-0357).^37^ The XRD results indicated that the samples FN3, FN5, FN7, and FN9 present a tetragonal crystalline FeNbO_4_ phase with cell parameters of *a* = 4.7, *b* = 4.7 *a*, *c* = 3.046, *α* = *β* = *γ* = 90.0°.^37,38^ The XRD patterns of FN3-9 are indexed well to the Rutile-type structure with 
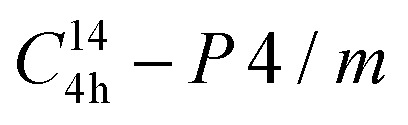
 nm raumgruppe.^37^ However, the sample of FN11 performed under high basic conditions (pH = 11) presents the tetragonal Fe_2_O_3_ crystalline phase (JCPDS No. 33–0664).^39^ Besides, the two peaks of FN9 at 24.2° and 30.08° are attributed to the (012) and (220) crystallographic planes of Fe_2_O_3_, indicating the presence of this crystalline phase in this sample. The sample FN9 shown a 93.6%, and 6.4% of FeNbO_4_ and Fe_2_O_3_ phases, respectively. This value is quantified by the RIR method and the MDI Jade software. This XRD result indicates that the increase in the pH of the media reaction in hydrothermal synthesis favours a transition crystalline phase between the FeNbO_4_ phase and the Fe_2_O_3_ phase.

**Fig. 2:**
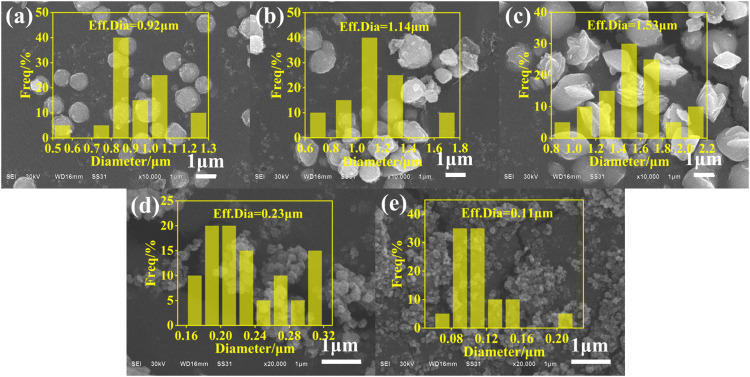
SEM image of different FeNbO_4_: (a) FN3, (b) FN5, (c) FN7, (d) FN9, and (e) FN11.

The morphology of the samples prepared in this work by hydrothermal synthesis (FN3, FN5, FN7, FN9, and FN11) has been studied by SEM and TEM analyses. Fig. 2a–e shows the SEM images of FN3, FN5, FN7, FN9, and FN11 samples, respectively. The SEM images of FN3, FN5 and FN7 samples show that under basic and neutral conditions the samples present an agglomeration of a laminar structure to micro-sphere particles (∼1 μm) due to their self-assembly.^40^Fig. 2a–e show that the particle size of the sample tends to increase with the increase in pH of the reaction medium under acidic conditions, and the surface tends to be smooth and sharp-edged. The size of 20 individual FNO particles was measured statistically using the Nano Measure 1.2 software. The average particle sizes of FN3 and FN5 are 0.92 μm and 1.14 μm, respectively. The FN7 (Fig. 2c) sample presents an average size of 1.53 μm, indicating an increase in the size of the particles when the pH in the medium of reaction was increased until pH = 7. However, when the reaction solution became alkaline, the samples have a heterogeneous morphology based on flocculated balls with a particle size of about 200 nm (Fig. 2d) for the FN9 sample and approximately 100 nm for the FN11 sample (Fig. 2e). These SEM results show a clear change in the morphology of FeNbO_4_ samples prepared in this work, indicating that the pH parameter in the hydrothermal synthesis affects the final morphology of the materials.^41,42^

**Fig. 3:**
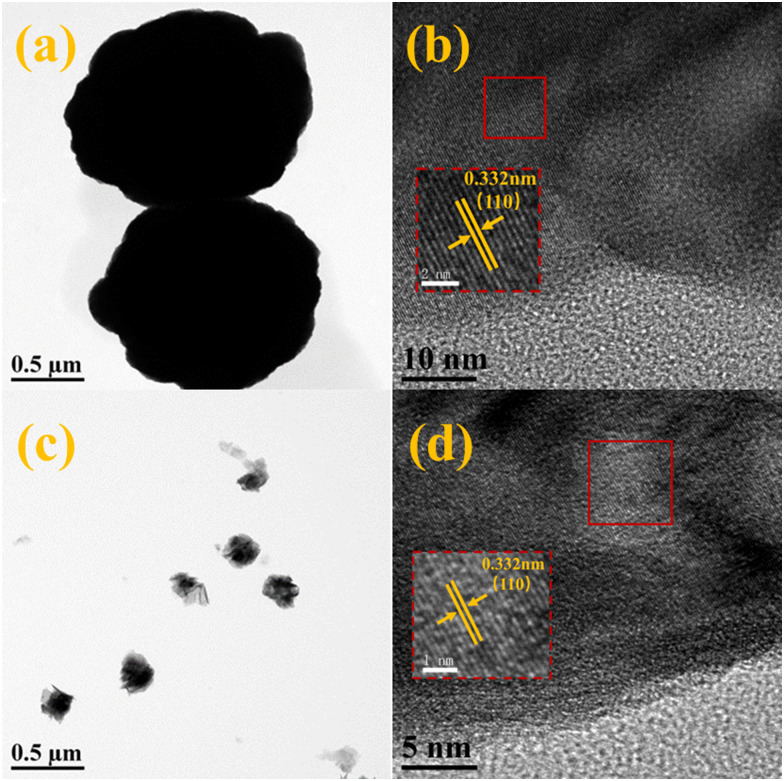
(a) TEM image of FN3. (b) HRTEM image of FN3. (c) TEM image of FN9. (d) HRTEM image of FN9.

The TEM images and HRTEM images of FN3 (Fig. 3a and b) and FN9 (Fig. 3c and d) samples are shown in Fig. 3. TEM analysis reveals that the FN3 sample presents a micro-spheric morphology and a particle size of around 1 μm (see Fig. 3a). The FN9 sample has a heterogeneous morphology based mainly on the spheric morphology with a particle size of about 200 nm (Fig. 3c). The results of TEM are in agreement with the SEM results. The HRTEM images (Fig. 3b and d) show the lattice fringes of the FN3 and FN9 samples, indicating high crystallinity of the prepared samples. The sample presents a lattice spacing can of 0.332 nm, which corresponds to the (110) crystal plane of the tetragonal FeNbO_4_ (JCPDS No. 16-0357). These results are in agreement with the XRD results obtained for this material.

**Fig. 4:**
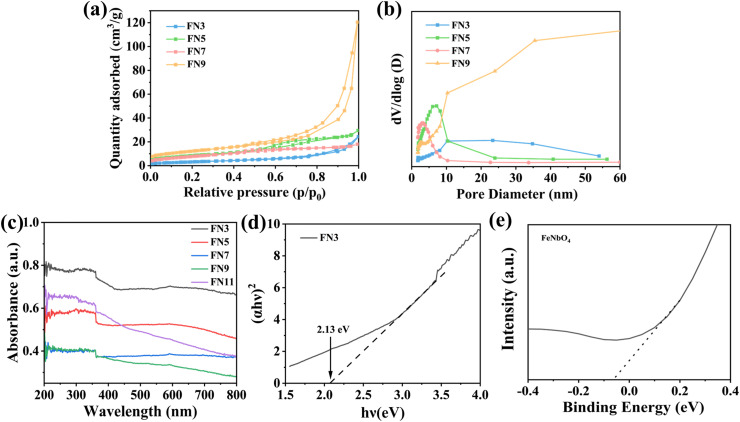
(a) N_2_ adsorption–desorption isotherms of different samples. (b) Pore size distributions. (c) UV-vis diffuse reflectance spectra of FN3. (d) (*αhv*)^2^*vs. hv* plots for FN3 samples. (e) Mott–Schottky plot of FN3.

The porous texture of the synthesized samples was investigated by N_2_ adsorption measurements (Fig. 4). The N_2_ adsorption–desorption isotherms of the samples prepared in this work (FN3, FN5, FN7, FN9) are shown in Fig. 4. Fig. 4 shows the isothermal adsorption curves of the samples; in the low-pressure region, an increase in the amount of N_2_ adsorbed is not observed. At higher relative pressures, an increase in the N_2_ adsorption is observed, showing pore filling. This effect is the characteristic of isotherms of type-III and hysteresis of type H3,^30^ indicating that FeNbO_4_ is a non-porous material. The FN9 sample presents the highest uptakes at low and high relative pressures than the FN3, FN5, and FN7 sample, indicating that the alkaline media in the synthesis favour the porosity in FeNbO_4_ materials. This fact was also evidenced by SEM and TEM analyses because the FN9 sample presents lower size particles with respect to FN3, FN5, and FN7 samples.

Concerning the textural properties shown in Table 1, the increase of alkaline conditions in the synthesis medium increases the surface area of the materials. In this sense, the specific surface area of FN9 was slightly larger than that of the other samples. This fact may be caused due to the small particle size of FeNbO_4_ under alkaline conditions as it was observed by SEM and TEM analyses. The pore sizes of FN3, FN5, and FN7 are decreased due to a smoother surface of the samples, as evidenced by the SEM analyses. However, the FN9 sample under alkali conditions presents a higher pore size. This fact evidences that the pH parameter affects the porosity and pore size of FeNbO_4_, as was observed by N_2_ adsorption–desorption isotherms.

**Table 1:** BET surface area and pore structure data of different FeNbO_4_ powders

Samples	BET surface area (m^2^ g^−1^)	Total pore volume (cm^3^ g^−1^)	Average porous size (nm)
FN3	12	0.036	12.7
FN5	31	0.043	5.9
FN7	29	0.022	3.9
FN9	44	0.12	17.1

The optical absorption properties of all samples were evaluated by UV-vis diffuse reflectance spectroscopy (Fig. 4c). In the figure, all samples showed a wide range of optical absorption. The intensity of optical absorption by the samples decreases with the preparation pH, with the FN3 sample exhibiting the maximum optical absorbance. It is also observed that the optical absorption becomes stronger when the pH increases to 11 (the FN11 sample). This may be due to the presence of a large amount of the Fe_2_O_3_ phase. The bandgap was calculated by the Tauc plot analysis in Fig. 4c, obtaining a value of 2.13 eV. This fact indicates that FeNbO_4_ is a semiconductor with an intermediate band gap, which is a very interesting property for sonocatalysis. The MS curve in Fig. 4e has a positive slope, indicating an n-type semiconductor characteristic.^43^ The flat band potential of the sample was determined to be −0.06 V (*vs.* Ag/AgCl) by extrapolating the linear part of the MS curve. Since the conduction band edge is usually close to the flat band potential, the band gap of the UV-vis absorption spectrum can be used to deduce the conduction band position of the FN3 sample. The bottom of the n-type semiconductor conduction band was typically about 0.2 V negative than the flat band potential, so the minimum value of the sample conduction band (CB) is estimated to be −0.26 V, and the maximum value of the valence band (VB) is estimated to be 1.87 V.^44^

**Fig. 5:**
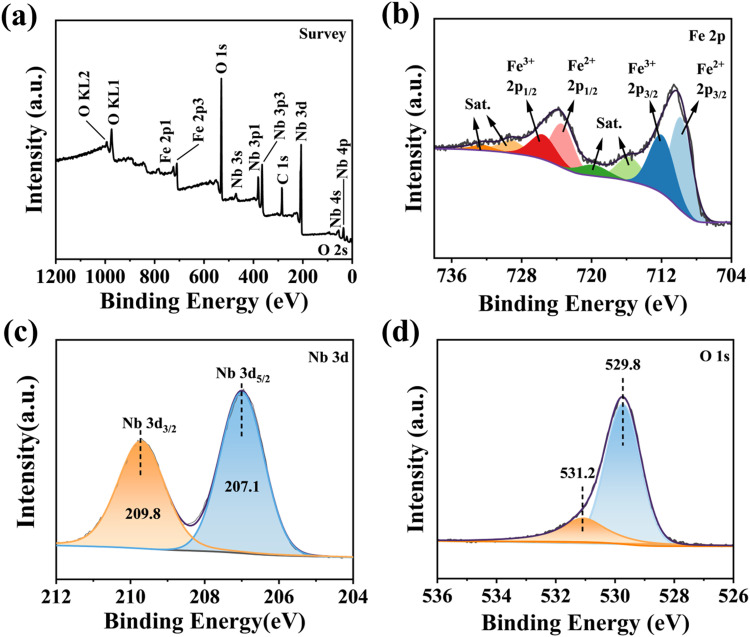
XPS spectra of the FN3 sample: (a) survey scan, (b) Fe 2p, (c) Nb 3d, and (d) O 1s.

X-ray photoelectron spectroscopy (XPS) analysis was used to determine the chemical composition and valence estates of the elements present in the prepared samples. The XPS survey showed that the main compositions of the FN3 sample were Fe, Nb, O and C elements (Fig. 5a), which contains 47.37% of O, 13.07% of Nb, 6.33% of Fe, and 33.23% of C. The high-resolution Fe 2p spectra (Fig. 5b) showed the presence of Fe^2+^ and Fe^3+^ in FeNbO_4_, two characteristic peaks located at 709.7 eV and 723.3 eV corresponded to 2p_3/2_ and 2p_1/2_ of Fe^2+^, respectively, and the two peaks at 715.6 eV and 729.1 eV were satellite peaks of Fe^2+^. In addition, two peaks attributed to 2p_3/2_ and 2p_1/2_ of Fe^3+^ were observed at 711.9 eV and 725.5 eV. Similarly, two peaks at 719.8 eV and 732.5 eV were satellite peaks of Fe^3+^.^18,45^ The results suggest that an oxidation/reaction process like-Fenton reaction may be occurring between Fe^3+^ and Fe^2+^.^46^ Nb 3d XPS spectra (Fig. 5c) show two peaks at 207.1 eV and 209.8 eV matching with Nb 3d_5/2_ and Nb 3d_3/2_, and this fact confirms the presence of Nb^5+^ in the samples,^47^ indicating that FeNbO_4_ was synthesized successfully. The O 1s XPS spectrum shows two peaks at 529.8 eV and 531.2 eV in Fig. 5d. The first one corresponds to O^2−^ ions at the lattice region and the second peak indicate oxygen deficiency regions.^43^

### 3.2 Ultrasound degradation

**Fig. 6:**
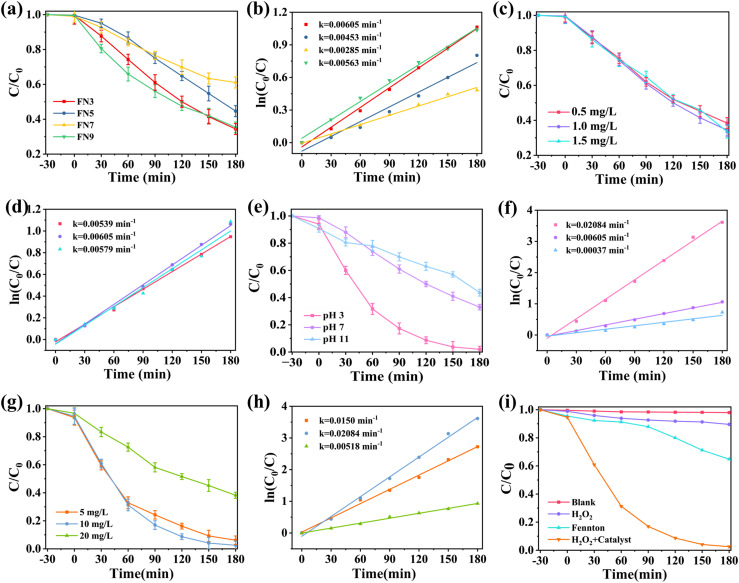
(a) Ultrasonic degradation of different FeNbO_4_ powders (catalyst dosage 1 g L^−1^, pH = 7, AO7 = 10 mg L^−1^). (b) The kinetic constants of different samples. (c) Ultrasonic degradation effect on different amounts of FN3 (pH = 7, AO7 = 10 mg L^−1^). (d) The kinetic constants at different catalyst amounts. (e) Ultrasonic degradation effect on different pH (FN3 = 1 g L^−1^, AO7 = 10 mg L^−1^). (f) The kinetic constants under different pH conditions. (g) Ultrasonic degradation effect on different concentrations of AO7 (FN3 = 1 g L^−1^, pH = 3). (h) The kinetic constants at different concentrations of AO7. (i) Comparison of dye degradation effects under different processing conditions (FN3 = 1 g L^−1^, AO7 = 10 mg L^−1^, pH = 7).

To evaluate the sonocatalysis activity of the prepared FeNbO_4_ samples, acid orange 7 (AO7) was targeted as the pollutant in this experiment. In this study, four parameters of sonocatalysis were studied (catalyst properties, catalyst concentration, effect of pH, and pollutant concentration). Fig. 6a shows the catalytic activity of the samples prepared in this work for their use in AO7 degradation under ultrasonic irradiation. The FN3 sample (65.59%) exhibits the best catalytic activity in a conversion of AO7 with a similar value to the FN9 sample (64.61%). The conversion value for the FN5 sample was 55.33% and the sample synthesised at a neutral pH exhibits a low catalytic activity value (38.9%). The catalytic test shows that the FeNbO_4_ sample presents activity in sonocatalysis and the condition of synthesis presents a great effect on the catalytic properties of the final material. The reaction kinetics of degradation is fitted as shown in Fig. 6b, and the degradation kinetics of all samples were consistent with first-order kinetics. These results show that the FN3 sample presents the best reaction rate constant 0.00605 min^−1^ with respect to other samples prepared in this work.

Fig. 6c shows the effect of different amounts of FN3 samples for catalytic degradation of AO7 under ultrasonic irradiation. In Fig. 6c, the catalytic conversion by three different concentrations was roughly the same, and the degradation rates were 61.55%, 65.59%, and 66.48%, respectively for 0.5 g L^−1^, 1 g L^−1^, and 1.5 g L^−1^. The increase in the catalyst concentration promotes the increase in the number of active factors on the catalyst surface, which will produce more active substances to participate in the degradation of AO7 solution and improve the degradation rate. 1 g L^−1^ of the catalyst is sufficient to provide sufficient active sites for promoting the formation of free radicals.^48^ The reaction rate constant of AO7 degradation is fitted as shown in Fig. 6d. The kinetics of the degradation reaction considering the effect of the catalyst concentration shows first-order kinetics.

To study the effect of the pH under ultrasonic catalysis performance, the pH values of the AO7 solutions were adjusted to 3, 7, and 11 by adding hydrochloric acid and sodium hydroxide solutions (catalyst dosage 1 g L^−1^ and dye concentration 10 mg L^−1^), respectively. As shown in Fig. 6e, the degradation rates of the dye solutions at pH = 3, 7, and 11 were 97.45%, 65.59%, and 56.08%, respectively, after 180 min of reaction. Fig. 6f shows that this parameter using the FN3 sample presents first-order kinetics. The high activity for the sample at acidic pH (pH = 3) is due to the high H^+^ concentration present in the solution. Then, the sulfo group in the azo dye is more easily removed, because of the high generation of radicals under this condition. Moreover, the electron-withdrawing properties of H^+^ make the unsaturated bond in the azo dye more easily broken, and thus the dye molecule is more easily decomposed. Therefore, the degradation effect of AO7 under acidic conditions is significantly higher than that under neutral or alkaline conditions.

Fig. 6g depicts the experimental results of ultrasonic catalysis when varying dye concentrations. The degradation rates of dye concentrations of 5 mg L^−1^ and 10 mg L^−1^ show high catalytic activity after 180 min of reaction, which are 93.84% and 97.45%, respectively. In this regard, the highest degradation rate was achieved when the dye concentration was 10 mg L^−1^. When the dye concentration was 20 mg L^−1^, the degradation rate decreased until 61.80% after 180 min of ultrasound irradiation. This effect might be due to a higher concentration, and the removal of AO7 was blocked due to the saturation of the adsorption and active sites. As it was mentioned for another parameters, the degradation kinetics of different dye concentrations (Fig. 6h) are consistent with the first-order kinetics.

Fig. 6i shows the comparison degradation effects under different processing conditions, such as (1) blank, (2) only H_2_O_2_, (3) Fenton condition, and (4) H_2_O_2_ + catalyst. The amount of the catalyst FN3 was 1 g L^−1^, the pH of the AO7 solutions was 7, and the solution concentration was 10 mg L^−1^. Without the presence of the catalyst (FN3) even using the H_2_O_2_ or Fenton process, there is no degradation of the AO7 dye. The incorporation of the catalyst (FN3) in the reaction media under sonification shows an increase in the degradation of AO7. When subjected to ultrasonic radiation, FeNbO_4_ and H_2_O_2_ were present together, the degradation effect was greatly improved. This effect may be due to the Fenton-like reaction introduced in the reaction system after the addition of H_2_O_2_.^49^ The addition of H_2_O_2_, which reacts with holes to form ˙OH, initiates the reaction process of FN3 as a semiconductor catalyst and reduces the electron–hole rate recombination.

**Fig. 7:**
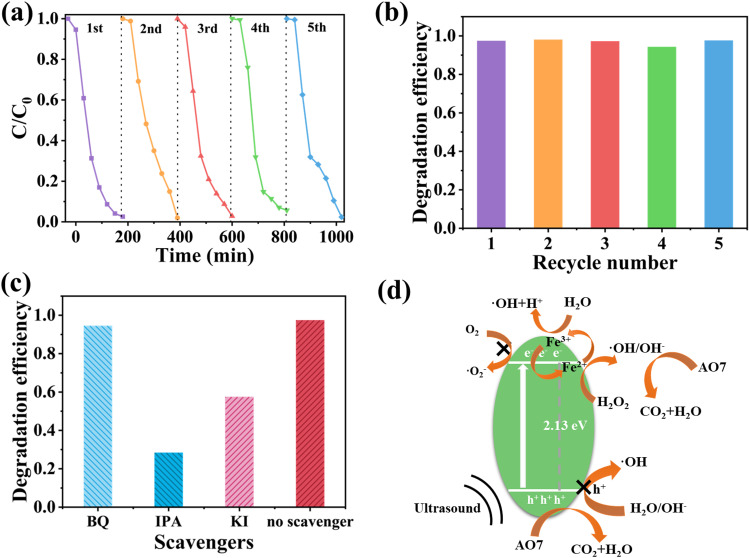
(a) Stability of FN3 in the ultrasonic catalytic experiment (FN3 catalyst dosage 1 g L^−1^, dye concentration 10 mg L^−1^, and pH = 3). (b) Degradation efficiency graph for cycling experiments. (c) Histogram of the ultrasonic degradation rate of AO7 by the FN3 sample after adding the capture agent. (d) Reaction mechanism of catalytic degradation of AO7 by FeNbO_4_ ultrasonic radiation combined with the Fenton-like reaction.

The recoverability and reusability of the sonocatalysts are important indicators for practical applications. Since the FN3 sample had the best sonocatalytic activity for AO7, the FN3 sample was chosen for the recycling experiment. The FN3 sample was recovered for recyclability experiments using the optimal experimental conditions because this catalyst exhibits the best sonocatalytic activity for AO7 and it is a pure material. The experimental results are shown in Fig. 7a and b. After 5 cycles, the degradation rate of the FN3 sample was still high, indicating that the FN3 sample has high recyclability.

In this study, the prepared catalysts were compared with those reported in the literature for the removal of AO7 at different types of energy sources. The results are shown in Table 2. First, FeNbO_4_ catalyst applications in ultrasonic catalysis are still rare. Second, in the case of ultrasonic energy, the removal efficiency from pure FeNbO_4_ was higher than other composites used in this application. The catalyst prepared in this study has a high removal efficiency of the azo dye under the excitation of ultrasound similar to another energy sources such as light, with makes this catalyst and this technology interesting for a future application.

**Table 2:** Comparison of the removal ratio of AO7 for different catalysts

Catalysts	Ultrasonic power (W)	Concentration (mg L^−1^)	Catalyst dosage (g L^−1^)	Degradation (%)	Time (min)	Ref.
FeNbO_4_	200	10	1	92	120	This work
CoFe_2_O_4_/rGO	350	10	0.08	90.5	120	50
CaMoO_4_	200	5	1	97.02 ± 0.65	120	51
CPP/TiO_2_/Ag	300	10	5	20	120	52
Ce_2_Sn_2_O_7_	240	10	1	58	120	6
Dy_2_Sn_2_O_7_/Sepiolite	240	10	1	84	120	53

The contribution of oxidative species in the AO7 removal process was studied by quenching experiments. In quenching experiments, KI, BQ (1,4-benzoquinone), and IPA (isopropanol) were used as scavengers of holes, ˙O_2_^−^ and ˙OH, respectively. Fig. 7c shows a slight inhibition with the addition of BQ in the removal of AO7, implying that ˙O_2_^−^ had a small influence on the catalytic efficiency. The degradation of AO7 was suppressed by KI and IPA, indicating that holes and ˙OH contribute to the elimination of AO7. The addition of IPA reduced the degradation rate to 28.41%, which had a great influence on the degradation effect. This indicates that the active species that play a leading role in the ultrasonic catalysis process are ˙OH and h^+^.

Based on the above experimental results, a possible ultrasonic catalysis mechanism may be proposed, as shown in Fig. 7d. The mechanical energy of ultrasonic wave acts on the liquid system and generates many extremely small cavitation bubbles. In the process of its formation to breaking, a large amount of energy released will generate local hot spots and be accompanied by sonoluminescence.^54^ The energy generated by ultrasound (*e.g.*, in the form of an optical cavity) causes an electron excitation in the semiconductor from the conduction band (CB) to the valence band (VB), generating the electron–hole pairs. The conduction band position of FN3 is −0.26 V, not close to the −0.33 V of O_2_/˙O_2_^−^, which means that it is impossible to produce ˙O_2_^−^, as it is in agreement with the results of quenching experiments. Indeed, the valence band position of FeNbO_4_ is 1.87 V, which is more negative than the potential of ˙OH/H_2_O (2.28 V).^55^ These results indicate that the h^+^ generated in the semiconductor FeNbO_4_ is not capable to generate the radical ˙OH. As shown in Fig. 6i, H_2_O_2_ contributed to the efficient degradation of AO7 by the sonocatalyst, which indicates that H_2_O_2_ plays a vital role in the degradation process. Quenching experiments confirmed the participation of ˙OH species in the reaction mechanism. The generation of the radical ˙OH could be explained because the presence of Fe^2+^ in the catalyst, as it is showed in XPS, might be reduced to Fe^3+^ by the e^−^ generated in the semiconductor. Then, the species Fe^3+^ could react with H_2_O_2_ to produce the ˙OH radical with enough oxidant capability to degrade the AO7 contaminant, following a Fenton-like reaction.Fe^3+^ + e^−^ → Fe^2+^Fe^2+^ + H_2_O_2_ → Fe^3+^ + ˙OH + OH^−^Fe^3+^ + H_2_O + *hν* → Fe^2+^ + ˙OH + H^+^

### 3.3 Discussion

In last few years, the AOP methods to degrade contaminants in water have attracted the attention of the scientific community. Moreover, the synthesis of novel routes to produce efficient catalysts to be used in AOP methods focusing on sonocatalysis and hybrid methodologies (photo-sonocatalysis) is one of the keys to apply this technology in real scale. In this regard, the scientific community has focused on the synthesis catalysts like TiO_2_, Fe/PS, TiO_2_-rGO, and ZnO, among others obtaining satisfactory results due to their use for pollutant abatement at the lab scale. Another alternative catalyst is the FeNbO_4_ that has been used for photocatalysis for pollutant abatement and for photoelectrochemical water splitting. In this study, we have synthesized the FeNbO_4_ catalyst using hydrothermal synthesis at a low temperature (200 °C) without templates and calcination step compared with the previous report in the literature, where they commonly use a temperature of 1000 °C. The synthetic method presented in this work is a simple hydrothermal method, which might be produced on a large scale. The FeNbO_4_ synthesized in this work shows for first time the degradation of AO7 by ultrasonic radiation, opening the door to use these materials as sonocatalysts. The sonocatalysis for remediation of contaminants in water is a promising green approach for their possible scalability. The results obtained in this work (97.45% (3 h)) are similar to the removal rates previously reported for AOPs using different catalysts in the literature as shown in Table 2. Furthermore, the catalyst exhibited excellent structural stability and catalytic activity under acidic conditions, highlighting its promising potential for the ultrasonically catalyzed degradation of organic compounds.

## 4.Conclusions

In this work, tetragonal FeNbO_4_ powders were prepared using a hydrothermal method (200 °C) with ferrous sulfate heptahydrate (FeSO_4_·7H_2_O) and niobium oxalate (Nb(HC_2_O_4_)_5_) as precursor materials under different pH reaction conditions. The characterization of the samples showed that the pH value of the synthesis medium has a great influence on the morphology and final properties of the FeNbO_4_ crystal. Regarding the catalytic activity of the samples prepared in this work for AO7 degradation, the best catalytic parameters are 1 g L^−1^ for the catalyst concentration, pH = 3 for the dye solution, and 10 mg L^−1^ for the dye concentration obtaining a high value of AO7 conversion (97.45%) under sonication irradiation. Experimental analysis by XPS shows that the Fenton-like reaction plays an important role in the ultrasonic degradation of acidic orange 7 by FeNbO_4_. The free radical trapping experiments show that ˙OH and h^+^ play a leading role in the degradation of organic matter. This study improved the synthesis method of FeNbO_4_ and provided the new idea and development direction for the application of niobate materials in the future.

## Author contributions

Min He and Defa Li: methodology, formal analysis, investigation, validation, and writing – original draft. Yu Liu: formal analysis and investigation. Feng Li: supervision, data curation, funding acquisition, and writing – review and editing. Taohai Li: conceptualization and writing – review and editing. Javier Fernández-Catalá: formal analysis and writing – review and editing. Wei Cao: supervision, writing – review and editing, and funding acquisition.

## Conflicts of interest

There are no conflicts to declare.

## Supplementary Material
